# Fasting mimicking diets: A literature review of their impact on inflammatory arthritis

**DOI:** 10.31138/mjr.30.4.201

**Published:** 2019-12-31

**Authors:** Aliki I. Venetsanopoulou, Paraskevi V. Voulgari, Alexandros A. Drosos

**Affiliations:** Rheumatology Clinic, Department of Internal Medicine, Medical School, University of Ioannina, Ioannina, Greece

**Keywords:** Fasting, metabolism, immunity, inflammation, rheumatoid arthritis

## Abstract

Fasting is an act of restricting, for a certain length of time, food intake or intake of particular foods, and has been part of religious rituals for centuries. Religions such as Christianity and Islam use this practice as a form of sacrifice, self-discipline, and gratitude. However, in the past decade, fasting has penetrated the mainstream as a diet trend. There are several ways of fasting; existing fast mimicking eating methods promise accelerated weight loss, and many more benefits: lower cholesterol, prevention of type 2 diabetes and a longer lifespan. Even more, it has been proposed that fasting can downregulate the inflammatory process and potentially be used as a treatment regimen for several diseases. Here, we review the effects of fasting on immune and inflammatory pathways. Also, we present current knowledge about the role of fasting in the activity of inflammatory arthritides with a focus on rheumatoid arthritis.

## INTRODUCTION

### Fasting methods

Fasting, throughout history and in almost all religions of the world, has long been promoted as a spiritual means that brings great mental and emotional health. However, nowadays, it is becoming an increasingly popular eating pattern, applied through well-known diet plans that mimic the fasting process with the aim of quick weight loss. There are many different methods used, such as intermittent fasting (IF) and fast mimicking diet (FMD), where fasting lasts from 12 hours to up to weeks at a time (*Table 1*). Fasting should not be confused to calorie restriction (CR), which is a whole different eating pattern. CR includes a consistent reduction on average daily caloric intake below to what is typical or habitual, without deprivation of essential nutrients. Many experiments have shown that CR feeding delays the onset of age-related disorders and may correlate to lifespan extension.^1,2,3^ Nevertheless, some studies have conflicting results^4^ that may be due to differences in dietary composition, and further investigation is needed.

**Table 1.** Most popular fasting methods.

Types	Fasting methods	Duration
IF*• cycling through periods of drastically cutting food intake with periods of healthy eating	**16/8 fasting diet** **5:2 fasting diet** **Alternate day fasting** **Warrior Diet** **One meal a day (OMAD)**	Healthy eating limited to a single 8-hour window every day Healthy eating for 5 days per week, and limiting calories to between 500 and 600 for 2 days a week Fasting every other day, and healthy eating during non-fasting days Fasting over a 20-hour window and then eating one large meal during a 4-hour evening window Fasting for 23 hours and eating daily calories during a 1-hour window
FMD**• cycling through periods of limiting calories intake, while providing essential nutrients such as vitamins and minerals		• Fasting 2-7 days every 15–365 days

* IF: Intermittent Fasting,

** FMD: Fast Mimicking Diet

### Fasting and health benefits

Studies have shown that fasting for short periods can increase metabolism.^5^ IF is assumed to influence the metabolic regulation via effects on (1) circadian biology, (2) the gastrointestinal microbiota, and (3) modifiable lifestyle behaviours.^5,6^ This hypothesis has driven research on animal and human subjects for decades, and has given significant evidence for the potential role of fasting on weight loss and even more on improved metabolism.^7^ A study by Dr. Longo et al.^8^ linked FMD to fat loss, as people in the fasting group, when completed three months of FMD, lost an average of 2.7 kg and experienced notable reductions in belly fat, blood sugar, and cholesterol levels. Also, IF optimizes autophagy, a process of self-repair through cellular regeneration, and thus may protect against mental decline and slow cellular aging. A study in mice found that short-term food restriction leads to a dramatic increase of autophagy in nerve cells,^9^ while animal models of vascular dementia that underwent alternate-day food deprivation for 12 weeks showed a significant reduction in oxidative damage to brain tissue and improved mental sufficiency.^10^ Furthermore, intermittent fasting purges precancerous or cancerous cells^6^ and recently a combination of FMDs with chemotherapy, immunotherapy or other treatments is proposed as a potentially promising strategy to improve the effects of cancer therapies.^11^ Given these results, in animals and clinical trials, researchers are now studying if and how FMDs affect lifespan, not only in obese, but also in non-obese people.

### Metabolism, immunity, and fasting

Metabolism and immune response present a tight interdependency, and today’s research has shown that glucose, amino acids (AAs), and fat acids (FAs) metabolism regulate leukocyte activation, subset differentiation, and function.^12^ Particularly, T cells during activation use mainly aerobic glycolysis converting glucose to lactate.

**Figure 1.**
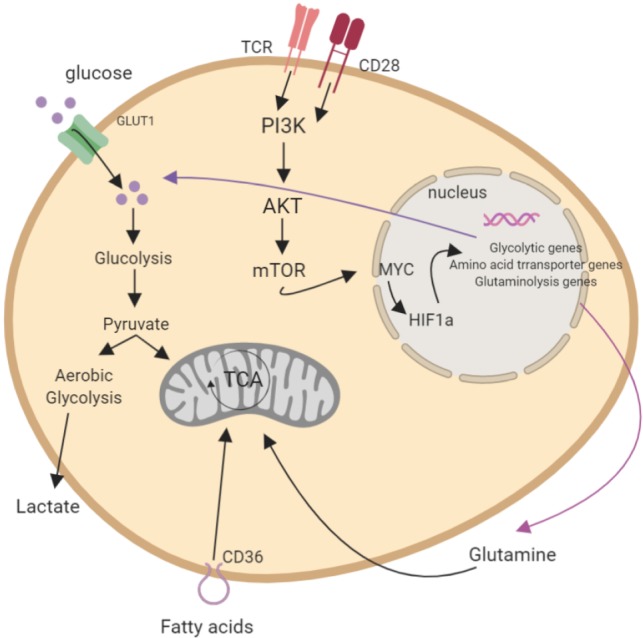
Cellular metabolism during T cells activation. During activation T cells convert glucose to lactate. This process is managed by the expression of glucose transporter 1 (Glut1). T-cell receptor (TCR) and CD28 increase the production of Glut 1 and through PI3K-Akt pathway Glut1 moves to T cell surface. Also, AAs enter leukocytes through glutamine transporters, and intracellular activate mTORC1 leading to the regulation of T cells. Finally, FAs diffuse across the plasma membrane or transport by FAT or CD36 receptors into the cytosol and inside the cell they ender TCA cycle.

This process is managed through increased glucose transporter 1 (Glut1) expression and surface localization (*Figure 1*). T-cell receptor (TCR) and CD28 co-stimulate and induce Glut1 upregulation, while the phosphoinositol-3 kinase (PI3K)-Akt pathway provokes the translocation of Glut1 from the cytoplasm to T cell surface. AAs are an essential fuel for activated T cells due to increased demands for protein synthesis and metabolites that enter into metabolic processes, such as the tricarboxylic acid (TCA) cycle. Upon activation, leukocytes increase the expression of many AAs transporters, including the leucine and glutamine transporters. Intracellular, AAs activate mechanistic target of rapamycin complex 1 (mTORC1), leading to the regulation of CD4T cell differentiation (Th1 and Th17) and CD8^+^ T cell response. Moreover, several studies indicate that de novo FAs synthesis is important for activation, proliferation, and differentiation of effector T cells. Development of CD8^+^T memory cells, as well as differentiation of CD4^+^ T regulatory (Treg) cells, is linked to FAs catabolism via transport of free-FAs into the cytosol and the mitochondria b-oxidation. Specific FAs diffuse across the plasma membrane into the cytosol, but most require transport by surface receptors such as FAs translocase (FAT) or CD36, and inside the cell they enter TCA cycle (*Figure 1*).^13^

Dysregulation of cell metabolism is implicated in the pathogenesis of autoimmune diseases.^14^ Systematic diseases like lupus, multiple sclerosis (MS), and inflammatory arthritis often present pathologic metabolic regulatory pathways leading to dysfunctional lymphocytes and disease progression. It is remarkable that, in rheumatoid arthritis (RA), a recent study showed that in contrast to healthy T cells, RA CD4 T cells fail to produce as much ATP and lactate due to the insufficient induction of 6-phosphofructo-2-kinase/fructose-2,6-bisphosphatase 3 (PFKFB3), a rate-limiting enzyme in the glycolytic pathway.^15^ Deficient activity of PFKFB3 shunts glucose towards the pentose phosphate pathway and generates increased levels of nicotinamide adenine dinucleotide phosphate (NADPH), which in turn eventually reduces intracellular reactive oxygen species (ROS). Reduced ROS production is associated with increased severity of joint inflammation.^16^ PFKFB3 also diminishes the activity of autophagy, but the RA T cells are unable to upregulate the autophagic process and are forced into apoptosis. Furthermore, studies have shown that T cells in RA patients present an accelerated aging phenotype due shortening of telomeres, loss of CD28, and reduced efficiency of DNA repair mechanisms.^17^ Although we are unable to distinguish whether glycolytic insufficiency precedes or follows the process of T-cell aging, it is inevitable that the lower ability of T cells to generate ATP makes them more sensitive to apoptosis and thus, cause a turnover toward a more lymphopenic host.

**Figure 2.**
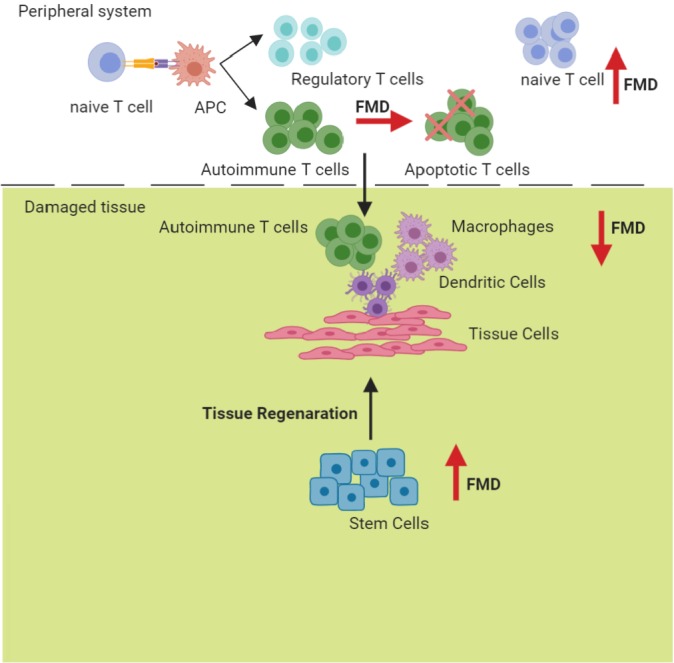
Alteration of autoimmune response through periodic FMD. Fasting through FMD causes apoptosis of the autoreactive T cells and leads to an increase of naïve T cells and Treg cells. In the lesion area, FMD promotes tissue repair as it stimulates precursor cells.

Fasting alters cellular metabolic pathways and affects immune function, through its impact on cell trafficking and proinflammatory cytokine expression. Studies indicate that IF during Ramadan attenuates inflammatory status of the body by decreasing markers of inflammation like C-reactive protein (CRP), tumour necrosis factor-alpha (TNF-α), interferon-gamma (INF-γ), leptin, interleukin 1 beta (IL-1β), and interleukin 6 (IL-6), but these alterations seem to be transient, returning to basal pre-Ramadan status shortly afterward fasting interruption.^18,19^ Furthermore, studies show that fasting modulates the IL-12/IL-10 cytokine balance^20^ and promotes the expression of endogenous IL-1 antagonists inducing IL-1 resistance.^21^ An animal study, using a murine model of MS, found that FMD cycles may be indeed effective in the reduction of specific inflammatory markers (INF-γ, IL-17, and TNF-α), and Th1 and Th17 cells.^22^ Moreover, previous findings indicate that FMD provokes apoptosis of the autoreactive T cells, leading to an increase of naïve T cells and Treg cells.^23^ Based on these results, a hypothetical simplified model of the effect of fasting, through FMD cycles, on the autoimmune response includes augmentation of Treg cells number, with simultaneous blockage of T-cell activation and promotion of T-cell death via apoptosis. In the lesion area, FMD may stimulate precursor cells and thus promote tissue repair (*Figure 2*).

### Fasting, rheumatoid arthritis, and other inflammatory arthritides

RA is an inflammatory-destructive joint disease. The development of RA is based on both genetic and environmental factors that affect the innate immune system leading to chronic inflammatory activity in the body.^24^ An interaction with a dysbiotic microbiome of the intestine, characterized by the loss of beneficial bacteria and concomitant increase in potentially pathogenic microbes, is associated with chronic inflammation in RA patients.^25^ Among other factors, overweight and obesity seem to have an adverse effect on the onset, progress, and disease disability.^26^

In recent years, numerous new therapeutic concepts have been developed. Still, response to treatment varies, and so far, obese RA patients show a higher degree of synovitis not only at disease onset but also after remission achievement, which affects the overall response to treatment.^27^ Thus, many patients often seek healing through alternative methods of which diet is an essential component. Interestingly, 20 to 50% of RA patients have tried dietary manipulation in an attempt to relieve their suffering.^28,29^

Current knowledge suggests that healthier nutrition by adjusting to a Mediterranean diet^30^ and a higher intake of fish^31^ is associated with a reduction in inflammatory activity, an increase in physical function, and improvement in RA patients’ vitality. Even more, supplementation with omega-3 polyunsaturated fatty acids (omega-3 PUFAs) reduces patients’ morning stiffness, painful joints, and Nonsteroidal anti-inflammatory drugs (NSAIDs) consumption.^32^

The role of fasting on RA disease activity has been studied thoroughly.^33^ Fraser et al. showed that patients who underwent 7-day subtotal fasting, with a limited amount of vitamin, mineral and carbohydrate supplementation, decreased CD4^+^ lymphocyte number and function, demonstrating a rapid immune suppression. Some clinical studies have linked fasting to the improvement of specific inflammatory markers such as IL-6, CRP, and erythrocyte sedimentation rate (ESR). At the same time, these patients present pain relief and reduction in Disease Activity Score 28 (DAS-28).^35,36,37^ No correlation between better disease outcome and intestinal flora alterations has been found in RA patients who follow a fasting diet plan, and further investigation is needed.^38,39,40^

However, inflammation returns when food is reintroduced, and symptoms flare up. Thus, fasting seems to have limited therapeutic value unless it is combined with other diet modifications, such a vegetarian diet.^41,42^ This approach has been studied by Kjeldsen-Kragh et al.^43^ in a single-blind controlled trial, where 53 patients with RA were randomly assigned to fasting or a control group. Patients of the diet group fasted for seven up to 10 days and afterward followed a vegetarian eating plan for 3.5 months. After 4 weeks, the diet group presented a significant improvement of the number of tender and swollen joints, pain score, and morning stiffness along with ESR, CRP, and other parameters. The beneficial effects were still present after two years of diet.^44^ Other trials have less convincing results, showing no significant impact of fasting followed by a Lacto-vegetarian diet.^45^ Yet, most of the existing trials indicate that commitment in a short fasting program followed by a modified diet provokes RA patients’ advantageous outcomes.^41,42,43,44,46^

Studies on the role of fasting in inflammatory arthritides, other than RA, are limited. Psoriatic arthritis (PsA), a T lymphocytes-mediated inflammatory disease that presents mainly with skin psoriasis and inflammation of the joints, and entheses, has been strongly linked to obesity.^47^ Weight reduction in obese patients may reduce the severe comorbidities associated with PsA and lead to a better overall outcome of the disease.^48^ A study by Damiani et al.^49^ showed that IF during Ramadan has beneficial effects on the activity of psoriasis disease expressed in the Psoriasis Area Severity Index (PASI). Finally, a recent report demonstrated a positive impact of IF on PsA patients, expressed by improvement in PsA disease activity scores, enthesitis, and dactylitis, regardless of the change in the patients’ weight.^50^ These findings support our current understanding over the role of fasting on immune pathways in inflammatory arthritides and promote interest for future investigation.

## CONCLUSIONS

Emerging research suggests that FMDs may lead to a healthier life and even aid cancer treatment. However, these claims remain controversial, and studies are primarily conducted in animal models. Fasting acts on cellular mechanisms and regulates the metabolism of immune cells. Thus, commitment to an eating pattern that includes a fasting component could suppress the inflammatory process. So far, most of the reported dietary interventions show beneficial effects on symptoms and disease progression in RA and PsA patients. Still, there is much to learn about fasting and the impact of different fasting patterns on non-obese and older patients, and more evidence is required before recommending any such eating regimens as supplemental “diet therapy” to patients with inflammatory arthritides.

## ABBREVIATIONS

AAs: Amino acids
CR: Calorie restriction
CRP: C-reactive protein
FAs: Fat acids
FAT: Fat acids translocase
FMD: Fast mimicking diet
Glut1: Glucose transporter 1
IF: Intermittent fasting
IL-1β: Interleukin 1 beta
IL-6: Interleukin 6
INF-γ: Interferon-gamma
MS: Multiple sclerosis
mTORC1: Mechanistic target of rapamycin complex 1
omega-3 PUFAs: Omega-3 polyunsaturated fatty acids
NADPH: Nicotinamide adenine dinucleotide phosphate
NSAIDs: Nonsteroidal anti-inflammatory drugs
PASI: Psoriasis Area Severity Index
PFKFB3: 6-phosphofructo-2-kinase/fructose-2,6-bisphosphatase 3
PI(3)K: Phosphoinositol-3 kinase
PsA: Psoriatic arthritis
RA: Rheumatoid arthritis
ROS: Reactive oxygen species
TCA: Tricarboxylic acid
TCR: T-cell receptor
TNF-α: Tumour necrosis factor-alpha
